# Normative data on regional sweat-sodium concentrations of professional male team-sport athletes

**DOI:** 10.1186/s12970-017-0197-4

**Published:** 2017-10-30

**Authors:** Mayur K. Ranchordas, Nicholas B. Tiller, Girish Ramchandani, Raj Jutley, Andrew Blow, Jonny Tye, Ben Drury

**Affiliations:** 1 Academy of Sport and Physical Acitivty, Sheffield Hallam University, Sheffield, S10 2BP UK; 2 Sport Industry Research Centre, Sheffield Hallam University, Sheffield, S10 2BP UK; 3 Precision Hydration, 43 Saffron Drive, Christchurch, BH23 4LR UK; 4 Hartpury University Centre, Hartpury, Gloucester, GL19 3BE UK

**Keywords:** Sweat, Sodium, Team-sport, American football, Basketball, Baseball, Soccer, Rugby (3–10)

## Abstract

**Background:**

The purpose of this paper was to report normative data on regional sweat sweat-sodium concentrations of various professional male team-sport athletes, and to compare sweat-sodium concentrations among sports. Data to this effect would inform our understanding of athlete sodium requirements, thus allowing for the individualisation of sodium replacement strategies. Accordingly, data from 696 athletes (Soccer, *n* = 270; Rugby, *n* = 181; Baseball, *n* = 133; American Football, *n* = 60; Basketball, *n* = 52) were compiled for a retrospective analysis. Regional sweat-sodium concentrations were collected using the pilocarpine iontophoresis method, and compared to self-reported measures collected via questionnaire.

**Results:**

Sweat-sodium concentrations were significantly higher (*p* < 0.05) in American football (50.4 ± 15.3 mmol·L^−1^), baseball (54.0 ± 14.0 mmol·L^−1^), and basketball (48.3 ± 14.0 mmol·L^−1^) than either soccer (43.2 ± 12.0 mmol·L^−1^) or rugby (44.0 ± 12.1 mmol·L^−1^), but with no differences among the N.American or British sports. There were strong positive correlations between sweat-sodium concentrations and self-reported sodium losses in American football (*r*_*s*_ = 0.962, *p* < 0.001), basketball (*r*_*s*_ = 0.953, *p* < 0.001), rugby (*r*_*s*_ = 0.813, *p* < 0.001), and soccer (*r*_*s*_ = 0.748, *p* < 0.001).

**Conclusions:**

The normative data provided on sweat-sodium concentrations might assist sports science/medicine practitioners in generating bespoke hydration and electrolyte-replacement strategies to meet the sodium demands of professional team-sport athletes. Moreover, these novel data suggest that self-reported measures of sodium loss might serve as an effective surrogate in the absence of direct measures; i.e., those which are more expensive or non-readily available.

## Background

There is a wealth of literature delineating the importance of electrolytes (i.e., sodium, potassium, calcium, magnesium, chloride) in the maintenance of plasma volume and cellular function during exercise (for review, see ref. [1, 2]). Sodium, specifically, is the major ion of the extracellular fluid, and has several important functions during exercise including fluid retention [3], and the generation of action potentials for muscle contraction [4]. Athletes with a history of heat cramp exhibit comparatively greater sweat-sodium concentrations relative to athletes with no history of cramp (54.6 ± 16.2 vs. 25.3 ± 10.0 mmol∙L^−1^) [5], although data on the causative relationship is equivocal [6]. Although rare, diets of inadequate sodium intake (below the recommended daily allowance of 2.4 g), combined with high sweat-sodium losses, may also result in symptoms comparable to that observed in overtraining syndrome; e.g., sleep disturbances and lethargy, which are mediated by an over-stimulation of the Renin-Angiotensin-Aldosterone system, causing sympathetic fatigue [7].

It is generally accepted that drinks containing carbohydrate and electrolyte formulations will promote better performance than water alone, particularly in athletes who exhibit substantial losses of fluid and sodium from prolonged or excessive sweating [3]. The guidelines pertaining to sodium replacement during exercise lasting more than 1 h are to consume 20–30 mmol^.^L^−1^ [3, 8]. However, there is large inter-individual variability in athlete sweat rate and composition [9], with differences in sweat composition persisting even when corrected for sweat rate, diet, and heat acclimation status [10]. Moreover, a retrospective analysis of 506 athletes from various sports (American football, baseball, basketball, soccer, tennis, cycling, running and triathlon), reported large age, sex, and body mass-related variations in sweat-sodium concentration [11] although sweat sodium concentrations for the individual sports were not reported. Others report large variability in the sweat-sodium losses of professional American football players, with values ranging from 15 to 99 mmol^.^L^−1^, despite no differences when players were grouped by playing position [12]. Collectively, these data highlight the lack of specificity in the current guidelines pertaining to sodium replacement during exercise, and exemplify the need for individualised strategies. Given the pivotal role of sodium in health and physical performance, it is also necessary to identify athletes who might be at a greater risk of excessive sodium losses during exercise. High-risk populations likely comprise those who compete in hot/humid conditions, for long time-periods, and those with combined high sweat-sodium concentrations and sweat-rates. Although there are previous studies on sweat-sodium concentrations in soccer [9, 13], rugby [14], and American Football [12, 15], there are no published studies making direct comparisons among professional sports with large cohorts. Moreover, because direct measures of sweat-sodium concentration require specialist equipment and expertise not readily available, the validity of athletes’ perceptions of sweat rates and sodium losses might serve as a useful tool in applied practice.

Accordingly, we performed a retrospective analysis on data collected from 696 professional male team-sport athletes, with several aims: 1) to report normative data on regional sweat sweat-sodium concentrations; 2) to compare sweat-sodium concentrations among sports; 3) to examine the relationships between sweat-sodium concentrations and various self-reported measures (self-reported sodium loss, self-reported sweat-rate, and self-reported cramping-frequency).

## Methods

### Participants

This retrospective analysis was approved by the institutional ethics review board. Data from 696 professional male team-sport athletes (soccer, *n* = 270; rugby, *n* = 181; baseball, *n* = 133; American football, *n* = 60; basketball, *n* = 52) were compiled after consultancy services were provided to clubs from North America (American football, basketball and baseball), and the United Kingdom (soccer and rugby) during a 4 year period (Dec, 2011 – Dec, 2015). As such, the participants in this study represent a convenience sample rather than those recruited from a random population of athletes. Prior to assessment, participants provided informed consent via digital signature administered using an online survey, and were asked to maintain a euhydrated state, to abstain from strenuous exercise for 24-h, and alcohol and caffeine for 12-h. Compliance was checked verbally on the morning of assessment and athletes were tested in an appropriate space at their respective training grounds.

### Experimental overview

Participants were required to attend their respective athletic institutions on a single occasion for assessment. During the visit, participants completed an online questionnaire on their perceptions of sweat-rate, sodium loss and cramping-frequency (see *Self-Reported Measures*), and underwent rested sweat-sodium tests via pilocarpine iontophoresis and subsequent conductivity analysis. Environmental conditions were not controlled but the collected sweat volumes met a specific pre-test criterion (see below).

### Sweat collection and analysis

**Fig. 1:**
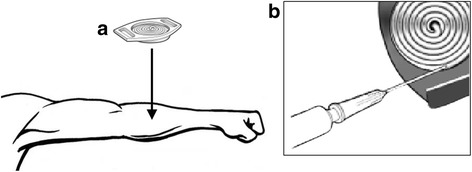
The pilocarpine iontophoresis method. An 85-μL sweat sample was collected through a bespoke plastic disc applied directly to the stimulated area of skin on the forearm (**a**). The sweat sample was then ejected from the duct under pressure using a blunt fill needle and syringe (**b**) and passed through a conductivity cell where it was analysed for sodium concentration

Sweat samples were collected from the participant following 5-min seated rest, using methods stated previously [16]. Briefly, sweating was induced via two stainless-steel electrodes and two iontophoretic discs applied to a localised area of skin on the left mid-forearm, through which a 1.5-mA iontophoretic current was applied for ~5-min (Webster Sweat Inducer, Wescor Inc., Utah, USA). The discs comprised a solid agar gel of 96% water, 0.5% pilocarpine nitrate and trace antifungal compounds. Prior to electrode placement, the skin was checked for breaks, fissures or inflammation, cleaned with alcohol to expel dirt and sweat [17], and swabbed with purified water. Following the removal of the iontophoretic discs, the stimulated area of skin was cleaned with purified water and blot-dried. An 85-μL sweat sample was collected through a bespoke plastic disc applied directly to the stimulated area of skin (Macroduct Sweat Collector, Wescor Inc.) through which sweat was collected via hydraulic pressure (Fig. 1). A full sample was usually obtained within 10–30-min, and samples of less than 85-μL were discarded.

The sweat sample was ejected from the collection disc using a blunt-fill needle and syringe, and passed through a conductivity cell (Sweat Chek™, Wescor Inc.), which calculated NaCl molarity from the sample conductivity. Each sample was passed through the analyser twice and the mean concentration recorded. Prior to each test, the conductivity cell was cleaned with deionised water and calibrated using a dummy sample of known sodium concentration. The techniques used in this study for the stimulation and collection of sweat samples have previously been validated against the Gibson and Cooke Gauze Technique [18], showing strong correlations with flame photometry (*r* = 0.99, ref. [19]) and quantitative pilocarpine iontophoresis (*r* = 0.99, ref. [16]). Moreover, the present technique of ion conductivity, when considered with four other methods of determining sweat-sodium concentration (ion chromatography, flame photometry, direct ion-selective electrode, indirect ion-selective electrode) showed excellent relative (ICC ≥ 0.99) and absolute (CV ≤ 2.6%) reliability [20]. In addition to being a validated method, pilocarpine iontophoresis is relatively quick to administer and is, therefore, appropriate when measuring large numbers of individuals in a short time period.

### Self-reported measures

Prior to sweat testing, participants completed an online survey in which they were asked three questions pertaining to perceived sweat rate, perceived sweat-sodium concentration, and perceived cramping-frequency. The questions (and answers) were phrased in the following manner: 1) "Would you classify your sweat rate as.." (*low, moderate, high, very-high*); 2) "How much sodium do you think you lose in your sweat?" (*low, moderate, high, very-high*); and 3) "How often do you suffer from muscle cramps" (*never, rarely, sometimes, often*). Prior to answering question two, participants were prompted to consider the frequency with which they observed white marks on their training apparel and/or sweat stinging their eyes. To determine between-day reliability of self-reported measures, 10 amateur male athletes completed the questionnaire on two occasions, separated by 1 week.

### Statistics

Statistical analysis was performed using SPSS 24.0 for Windows (IBM, Chicago, IL). Differences in sweat-sodium concentrations among sports were compared using one-way ANOVA. A Spearman’s rank-order correlation was run to assess the relationship between sweat-sodium concentration and self-report sodium loss, sweat-sodium concentration and self-reported cramping-frequency, and self-reported cramping-frequency and self-reported sweat rate. Between-day reliability of self-reported measures was assessed using intra-class correlation and Cronbach Alpha. Cramping-frequency was coded using the following scale: never (1), rarely (2), sometimes (3), often (4), while sweat rate and self-reported sodium loss were coded using the following scale: low (1), moderate (2), high (3), very high (4). Cronbach Alpha scores were interpreted as ‘good’ when values were >0.8, and ‘excellent’ when >0.9 [21]. The significance level was set at *p* < 0.05 and all data are presented as mean ± standard deviation.

## Results

### Sweat-sodium concentrations

**Fig. 2:**
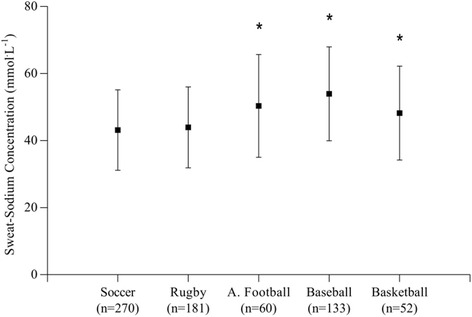
Sweat-sodium concentrations for professional soccer (*n* = 270), rugby (*n* = 180), American football (*n* = 60), baseball (*n* = 133) and basketball (*n* = 52). Individual means of sweat-sodium concentrations in American football, baseball and basketball were significantly greater than the individual means of soccer and rugby, but with no differences within the two sub-groups. * Significantly greater than soccer and rugby (*p* < 0.05)

Mean data for sweat-sodium concentrations in all sports are shown in Table 1 and Fig. 2. The assumption of homogeneity of variance, as assessed by Levine’s test, was violated (*F*(4691) = 4.161, *p* = 0.002), and so a Welch F-test was conducted with a Games-Howell post-hoc comparison. Sweat-sodium concentrations were statistically different among sports (*Welch’s F* (4, 192) = 17.024, *p* < 0.001). Post-hoc comparisons showed that the individual means of American football, baseball and basketball (i.e., data collected in the United States) were significantly greater than the individual means of soccer and rugby (i.e., data collected in the United Kingdom; *p* < 0.05). There were no differences in sweat-sodium concentrations among American football, baseball and basketball (*p* > 0.05), or between soccer and rugby (*p* > 0.05). Sweat-sodium concentration ranged from 17 mmol^.^L^−1^ (in soccer) to 92 mmol^.^L^−1^ (in baseball).

**Table 1:** Mean sweat-sodium concentrations for five professional team sports

	Mean		SD	Lower	Upper
	(mmol∙L^−1^)			(mmol∙L^−1^)	(mmol∙L^−1^)
Soccer (*n* = 270)	43.2	±	12.0	17	85
Rugby (*n* = 181)	44.0	±	12.1	20	83
A. Football (*n* = 60)	50.4	±	15.3*	21	84
Baseball (*n* = 133)	54.0	±	14.0*	29	92
Basketball (*n* = 52)	48.3	±	14.0*	29	89
Overall mean (*n* = 696)	44.4	±	13.6	23.2 ± 5.5	86.6 ± 3.8

Data are means ± SD. *significantly different to rugby and soccer *p* < 0.05

### Self-reported measures

**Fig. 3:**
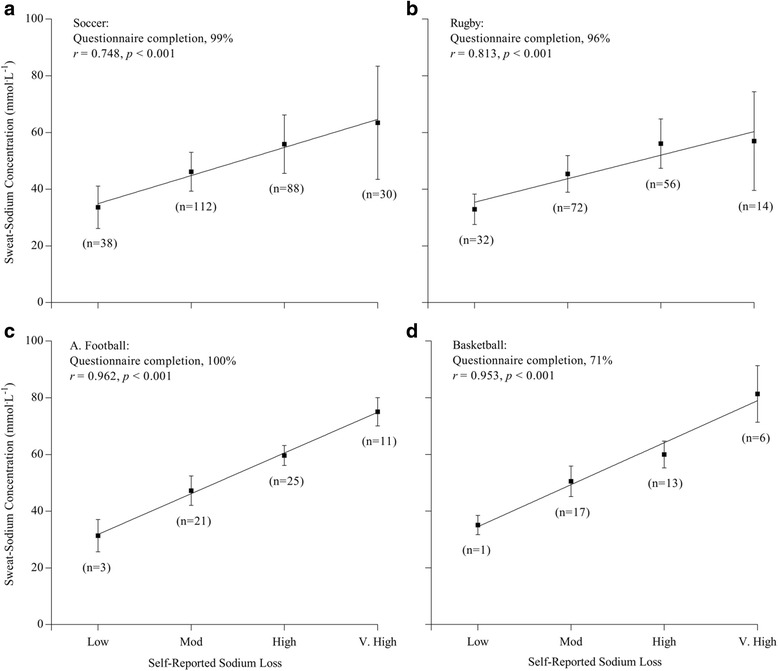
Correlation coefficients for self-reported sodium loss and regional sweat-sodium concentrations for professional soccer (panel **a**; *n* = 270), rugby (panel **b**; *n* = 180), American football (panel **c**; *n* = 60), and basketball (panel **d**; *n* = 52)

Reliability of self-reported measures is shown in Table 2. There were no systematic differences in self-reported measures when 10 participants completed questionnaires 1 week apart (*p* > 0.05). Moreover, the between-occasion reliability was good - excellent (all Cronbach Alpha >0.8; all ICC > 0.7). Thus, the perceptual measures were likely sufficiently reproducible to detect relationships between athlete perceptions and quantitative variables. The correlation coefficients for sweat-sodium concentration and self-reported sodium loss, sweat-sodium concentration and self-reported cramping-frequency, and self-reported sweat rate and self-reported cramping-frequency are shown in Table 3. Questionnaire completion rates and individual responses for self-reported sodium loss by sport are shown in Fig. 3. There was a positive correlation between sweat-sodium concentration and self-reported sodium loss (*r*_*s*_ = 0.818, *p* < 0.001). Specifically, significant correlations were observed for American football (*r*_*s*_ *=* 0.962, *p* < 0.001), basketball (*r*_*s*_ = 0.953, *p* < 0.001), rugby (*r* = 0.813, *p* < 0.001), and soccer (*r*_*s*_ = 0.748, *p* < 0.001) (*Note:* due to technical issues, self-reported data were not collected for the baseball cohort). There was a weak positive correlation between sweat-sodium concentration and self-reported cramping-frequency in rugby (*r*_s_ = 0.184, *p* = 0.015), however, the correlations for sweat-sodium concentration and self-reported cramping-frequency in all other sports were weak and non-significant (*p* > 0.05). There were also no significant correlations between self-reported cramping-frequency and self-reported sweat rate in any sport.

**Table 2:** Between-day reliability of self-reported measures

	Trial 1			Trial 2			Cronbach Alpha	ICC
Q1. Sweat-Rate	2.8	±	1.0	2.8	±	1.0	1.0	1.0
Q2. Sodium loss	2.4	±	0.7	2.2	±	0.8	0.8	0.7
Q3. Muscle Cramp	2.1	±	0.6	2.2	±	0.6	0.9	0.9

*ICC* intra-class correlation coefficient. Data are means ± SD, *n* = 10

**Table 3:** Correlation coefficients for sweat-sodium concentrations and self-reported measures

	[Na^+^]- self-reported sodium loss			[Na^+^]- self-reported cramping			Self-reported cramping- self-reported sweat- rate	
	*r*_*s*_	*p*		*r*_*s*_	*p*		*r*_*s*_	*p*
Soccer (*n* = 270)	0.748	<0.001	**	0.055	0.371		0.049	0.421
Rugby (*n* = 181)	0.813	<0.001	**	0.184	0.015	*	−0.030	0.689
A. Football (*n* = 60)	0.962	<0.001	**	0.114	0.387		0.189	0.147
Basketball (*n* = 52)	0.953	<0.001	**	0.095	0.577		0.308	0.064

[Na+], sweat-sodium concentration. Data are means ± SD. Significantly correlated * *p* < 0.05; ** *p* < 0.01. mxf: Self-reported data were not collected for the baseball cohort

## Discussion

The aims of this study were to report normative data on regional sweat-sodium concentrations of professional male team-sport athletes, to compare sweat-sodium concentrations among sports, and to examine the relationships between sweat-sodium concentrations and various self-reported measures. The novel findings are that professional athletes competing in American football, baseball and basketball exhibit significantly greater regional sweat-sodium concentrations than athletes competing in soccer or rugby. Furthermore, we report strong positive correlations between sweat-sodium concentrations and self-reported sodium losses across all sports.

An earlier retrospective analysis of team-sport athletes found that the sweat-rates of baseball, basketball and American football players were greater than that reported for soccer [11], although the sweat-sodium concentrations of each independent sport were not reported. The present study is the first, therefore, to report significant differences in the sweat-sodium concentrations of professional athletes training in North America (American football, basketball, baseball) relative to those training in the United Kingdom (soccer and rugby). The differences observed are unlikely due to the greater heat-acclimatisation status of athletes in North American sports, since acclimatisation has been shown to reduce the sweat-sodium concentration [22], due primarily to the reabsorption of sodium through the sweat duct [23]. Moreover, the sports that were evaluated comprise athletes from a myriad of ethnic backgrounds, which discounts ethnicity as a primary explanation for the differences observed between groups. Other factors including age, body-composition, training status, and sweat rate have no association with sweat-electrolyte content [24], although this latter marker has been contested [25]. Aldosterone is supressed in response to a sodium-rich diet, which can account for higher sweat-sodium concentrations [26, 27]. Given that dietary sodium, as assessed via urinary excretion, is higher in the United States compared to the United Kingdom [28], the sweat-sodium concentration differences between groups might be related to dietary sodium, although, this was not currently measured.

In 696 professional male team-sport athletes, we report regional mean sweat-sodium concentrations of 46.4 ± 13.6 mmol^.^L^−1^, which is in accordance with values previously reported for elite male athletes (48.2 ± 18.1 mmol^.^L^−1^) [11]. Furthermore, the values we report are congruent with values observed in soccer (43.2 versus 42.5 mmol^.^L^−1^, ref. [29]), rugby (44.0 versus 41.9 mmol^.^L^−1^ ref. [14]), and American Football (54.0 versus 50.3 mmol^.^L^−1^, ref. [14]). Although Baker et al. [11] report mean data encompassing several sports (including baseball and basketball), data regarding regional sweat-sodium concentrations for individual sports were not reported. As such, to our knowledge, we are the first to report normative values for professional baseball and basketball.

We also observed a strong positive correlation between sweat-sodium concentration and self-reported sodium loss. This suggests that self-reported measures of sodium loss might be a good predictor of actual sodium loss. The relationship is likely to be attributable, at least in part, to greater accuracy in the reporting of these subjective measures due to the visual prompting (e.g., salt residue on training apparel) and the recall of sensory experiences (e.g., stinging eyes and salt-taste in sweat) utilised in the questionnaire. Although we did not measure sweat rates in our study, others have found significant correlations for self-reported sweat rates with actual sweat rates in male basketball players [30]. Collectively, these findings suggest that both self-reported sodium losses and self-reported sweat rates might serve as an effective surrogate in the absence of more direct measures.

In contrast, we found that only the rugby sample exhibited a weak positive correlation between sweat-sodium concentration and self-reported cramping-frequency (*r*_s_ = 0.184, *p* = 0.015). Given the poor correlation coefficient, and the fact that no such correlations were observed in soccer, American football, or basketball, the relationship between sweat-sodium concentration and self-reported cramping-frequency is unlikely to be meaningful. Moreover, the causes of cramp are multi-factorial [6] and, therefore, evidence for a causative relationship between electrolyte depletion and cramping remains equivocal.

### Practical implications

Based on the data collected in this study, we report large inter-sport and inter-athlete variations in sweat-sodium concentration (range, 17–92 mmol^.^L^−1^). Moreover, athletes competing in baseball, American football, and basketball, respectively, exhibit higher sweat-sodium concentrations relative to rugby and soccer. Since the sodium content of many commercial sports drinks ranges from 10 to 25 mmol^.^L^−1^ [31, 32], it is likely that the drinks habitually consumed by competitive athletes contain sodium concentrations that are insufficient to replace sodium losses. Our findings, therefore, suggest that sodium concentrations substantially greater than 25 mmol^.^L^−1^should be consumed, but concentrations greater than 50 mmol^.^L^−1^ should be avoided as this will compromise drink palatability [33]. Moreover, special consideration should be given to the fluid volume and sodium concentration required to facilitate recovery following high-intensity or prolonged exercise. Many team-sport athletes have dense fixture lists and daily training and, therefore, normal eating and drinking practices are likely to be insufficient to replenish fluid and electrolyte balance for full recovery [34]. Therefore, aggressive fluid and electrolyte intake should be encouraged, particularly for athletes exhibiting the greatest sweat-sodium losses.

It also appears that self-reported sodium losses might be useful in estimating sweat-sodium concentrations when the latter cannot be measured directly. Therefore, athletes are encouraged to report their perceived sodium losses during exercise, making use of appropriate visual prompts (e.g., identifying sodium residue on training apparel) and sensory recall (e.g., experiencing the discomfort of stinging eyes, tasting salt in sweat).

### Technical considerations

There are several important considerations that should be made when interpreting the data presented in this study. First, it is well-established that there exist regional differences in sweat-electrolyte content [35], and regional sweat collection techniques have been shown to overestimate sweat-sodium concentrations when compared to whole-body values [36–38]. It is, therefore, common to collect sweat samples from various body locations (e.g., forearm, upper-back, chest, thigh) and combine measures into an arithmetic mean, or use a weighting-factor to account for regional differences. Regression equations have also been used to adjust raw values for regional sweat-sodium data; however, such correction equations were derived from sweat-patch tests, whereas the published literature has employed a range of different techniques in the collection of regional sweat-sodium concentrations, which might compromise the validity of their reported measures. Since the techniques employed presently are reliable and have been adequately validated (see *Methods*), we opted to report data for regional concentrations. Care should be taken, however, when extrapolating our findings to studies that have used different techniques. Second, the purpose of this paper was to report normative data on regional sweat-sodium concentrations in a large cohort of professional team-sport athletes. In addition to data on sweat-sodium concentrations, it should be noted that effective fluid and electrolyte replacement strategies cannot be determined without additional measures of whole-body sweat-rate; however, this can be estimated from pre-to-post-exercise changes in body mass. Third, heat acclimation status has been shown to influence sweat-sodium concentration [22], but could not be accounted for in the present observational study. Finally, this study induced the sweat response via pilocarpine iontophoretic discs applied to the skin, which has been shown to reflect heat- and exercise-induced sweating capacity, but is yet to be validated against the maximal and whole-body sweat response [39].

## Conclusions

In conclusion, we report normative data on the sweat-sodium concentrations of 696 professional male team-sport athletes using the pilocarpine iontophoresis technique. The main findings were that sweat-sodium concentration was significantly higher in the team-sport athletes competing in North America relative to those competing in the United Kingdom. Moreover, we present novel data pertaining to the validity of self-reported sodium losses in estimating sweat-sodium concentrations and, therefore, encourage the reporting of such perceptual data in the absence of direct measures. The data herein can be used by sports science/medicine practitioners to generate bespoke hydration and electrolyte-replacement strategies for professional team-sport athletes. Future studies should aim to report normative values for female, youth, and solo-sport athletes, in addition to assessing the validity of self-reported measures in non-professionals.
